# Neurohybrid Memristive CMOS-Integrated Systems for Biosensors and Neuroprosthetics

**DOI:** 10.3389/fnins.2020.00358

**Published:** 2020-04-28

**Authors:** Alexey Mikhaylov, Alexey Pimashkin, Yana Pigareva, Svetlana Gerasimova, Evgeny Gryaznov, Sergey Shchanikov, Anton Zuev, Max Talanov, Igor Lavrov, Vyacheslav Demin, Victor Erokhin, Sergey Lobov, Irina Mukhina, Victor Kazantsev, Huaqiang Wu, Bernardo Spagnolo

**Affiliations:** ^1^Lobachevsky State University of Nizhny Novgorod, Nizhny Novgorod, Russia; ^2^Department of Information Technologies, Vladimir State University, Murom, Russia; ^3^Neuroscience Laboratory, Kazan Federal University, Kazan, Russia; ^4^Department of Neurologic Surgery, Mayo Clinic, Rochester, MN, United States; ^5^Laboratory of Motor Neurorehabilitation, Kazan Federal University, Kazan, Russia; ^6^Kurchatov Institute, Moscow, Russia; ^7^CNR-Institute of Materials for Electronics and Magnetism, Italian National Research Council, Parma, Italy; ^8^Center for Technologies in Robotics and Mechatronics Components, Innopolis University, Innopolis, Russia; ^9^Cell Technology Group, Privolzhsky Research Medical University, Nizhny Novgorod, Russia; ^10^Institute of Microelectronics, Tsinghua University, Beijing, China; ^11^Dipartimento di Fisica e Chimica-Emilio Segrè, Group of Interdisciplinary Theoretical Physics, Università di Palermo and CNISM, Unità di Palermo, Palermo, Italy; ^12^Istituto Nazionale di Fisica Nucleare, Sezione di Catania, Catania, Italy

**Keywords:** memristor, neuronal culture, spiking neural network, microfluidics, biosensor, neuroprosthetics

## Abstract

Here we provide a perspective concept of neurohybrid memristive chip based on the combination of living neural networks cultivated in microfluidic/microelectrode system, metal-oxide memristive devices or arrays integrated with mixed-signal CMOS layer to control the analog memristive circuits, process the decoded information, and arrange a feedback stimulation of biological culture as parts of a bidirectional neurointerface. Our main focus is on the state-of-the-art approaches for cultivation and spatial ordering of the network of dissociated hippocampal neuron cells, fabrication of a large-scale cross-bar array of memristive devices tailored using device engineering, resistive state programming, or non-linear dynamics, as well as hardware implementation of spiking neural networks (SNNs) based on the arrays of memristive devices and integrated CMOS electronics. The concept represents an example of a brain-on-chip system belonging to a more general class of memristive neurohybrid systems for a new-generation robotics, artificial intelligence, and personalized medicine, discussed in the framework of the proposed roadmap for the next decade period.

## Introduction

The growing demand in miniature and energy-efficient electronic interface with bioelectrical activity for personalized medicine and other related products essentially depends on development of biohybrid electronic technologies (Vassanelli and Mahmud, 2016). The emergence of new technologies for creating thin-film sensors and non-invasive signal processing systems ensures the development of fundamentally new approaches to solve the problems of recording activity signals of brain, heart, and muscles, as well as skin condition in the form of wearable systems for processing and diagnostic. Such bio-compatible microelectronic systems, along with new biotechnologies, may provide a breakthrough in the field of neuroprosthetics with an important competitive advantage: a miniature bioelectrical sensor based on micro- and nanostructures with an option to store and process signals in multiple manners, including feed-forward approach and feedback loops, may serve as an active neurointerface for intelligent control and management of neuronal structures.

A wide variety of neuroprosthetic technologies have emerged recently from prosthetic arms (Fukuma et al., 2016; Petrini et al., 2019b) and legs (Petrini et al., 2019a) to prosthetic hearing (Rouger et al., 2007) and vision (Fernandez, 2018). Some of limb prostheses were non-invasively controlled by electrical signals from the muscles electrical activity (myograms) or electrical activity of selected areas of the motor cortex. The most promising bionic technologies are aimed at creating prosthetic devices controlled by the electrical activity of neurons via specialized arrays of electrodes implanted into neural tissue. In order to provide sensory feedback, additional arrays can also be implanted in somatosensory areas of the cerebral cortex or afferent systems of the spinal cord. The implantation of sensor chips into the visual cortex or a retina of an eye is becoming a serial operation today (Chuang et al., 2014). Cochlear implants are used by hundreds of thousands of patients around the world (NIH, 2016). Another promising direction is neurohybrid computing systems with living neural cells cultured in a nutrient medium *in vitro* and, after the maturation and formation of a large number of synaptic connections between cells, implemented to control an external robotic device or solve the complex sensory-cognitive task (e.g., pattern recognition). These devices also called neuroanimats in the literature (Xydas et al., 2008).

Another modern technology, memristors, possess the unique property of non-linear resistive memory and could serve as analog information processing systems with a neuron-like structure, as well as an electrophysiological activity sensor with capacity of simultaneous accumulation and non-volatile storage. Further development of memory-embedded sensors (Tzouvadaki et al., 2015; Doucey and Carrara, 2019) and neurohybrid systems, including neuroprostheses based on the integration of memristive and microelectrode CMOS technologies, as well as spiking neural network (SNN) architectures, will ensure the processing and real-time classification of electrophysiological and other analog signals, related to the activity of biological neuronal networks. Potential applications of this technology may target *in vivo* testing of pharmacological effects, biosensors and detectors of electromyography (EMG) signals, as well as muscle force extraction for various technical systems (smart tissue, wearable electronics, smart wheelchairs, cyber-physical suits, and vehicles). Most challenging problems are currently related to the application of implantable and non-implantable machine-to-nervous-system interfaces and neuroprostheses for correcting and restoring cognitive abilities, complex motor patterns like locomotion, and vision.

In this perspective, we discuss the main challenges associated with development of compact multifunctional neurohybrid systems for the bidirectional interface of living biological systems and memristive electronics combined with microelectrode and microfluidic systems. As compared to the previous works (Vassanelli and Mahmud, 2016; Chiolerio et al., 2017) focused on general trends and approaches for interfacing between neuronal and extrinsic/intrinsic neuromorphic systems, here we provide a comprehensive analysis of the implementation of a CMOS-integrated hybrid system based on scalable memristive devices and arrays back-end-of-line or monolithically integrated with CMOS circuits, analog signal processing on CMOS chips with memristive and microelectrode arrays. Specialized memristive neural architectures are proposed to implement functional abilities of some regions of the brain and nervous system. A roadmap of research and development in the field of memristive neuromorphic and neurohybrid systems has been for the first time presented and discussed in this manuscript in the context from state-of-the-art tasks to future challenges (until 2030).

It is worth noting here that memristors provide only one of the possible options for creating biomimetic electronic systems for neural interfaces. In particular, the neuromorphic function has also been demonstrated in colloidal nanomaterials or networks of nanowires (O’Kelly et al., 2016; Manning et al., 2018) and organic electrochemical transistors (Gkoupidenis et al., 2015, 2017; Tarabella et al., 2015; Battistoni et al., 2019b). Certain advantages of such materials over CMOS architectures have been discussed in recent reviews (Inal et al., 2018; Rivnay et al., 2018; van De Burgt et al., 2018; Ling et al., 2020) and mainly related to the flexibility and mechanical property match with neural tissue, the lower impedance, and current densities. Nevertheless, they are outside the scope of this perspective, and we will limit ourselves only to the CMOS-compatible approaches that are ready for the integration into existing technological workflows dedicated to practical applications. The focus on metal–oxide memristive electronics will allow going beyond the traditional neuromorphic chips as parts of neurohybrid systems (Hogri et al., 2015; Boi et al., 2016; Buccelli et al., 2019).

## Memristive Neurohybrid Chip: Concept and Challenges

According to the general definition (Vassanelli and Mahmud, 2016; Chiolerio et al., 2017), the neurohybrid system provides an interaction between biological (neuronal) and artificial elements in the open- or closed-loop manner. Despite the large number of available examples, they usually reflect different sides of such interaction and primary confirm some level of connectivity between biological and artificial systems. A functional interface between simple living being (slime mold) and memristor devices has been reported (Adamatzky et al., 2012) and, recently, the possibility of direct synaptic coupling of neuron cells from the rat cortex through a memristive device has been demonstrated (Juzekaeva et al., 2019). Future implementation of this approach requires the development of interrelated solutions at all levels, using both existing and emerging technologies in a single conceptual map matching the requirements for compactness, performance, energy efficiency, speed, reliability, and safety. In this paper, we analyze such solutions within the framework of a single concept of a neurohybrid CMOS chip that implements a compact interface between the biological (neuronal) system and the electronic subsystem.

**FIGURE 1:**
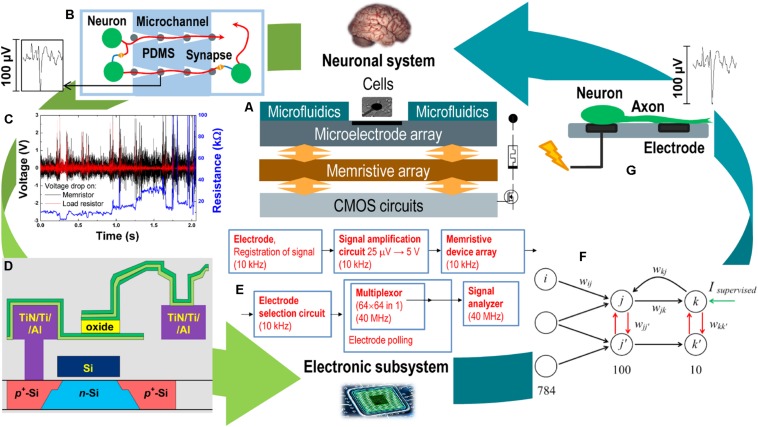
Memristive neurohybrid chip. **(A)** Schematic representation of the neurohybrid chip composed of a neuronal system (the brain cellular culture grown on MEA) and an electronic subsystem represented by the mixed analog–digital circuits coupling microelectrode arrays, memristive devices, and intrinsic neuromorphic systems. **(B)** The sketch of a spatially ordered neuronal culture with individual axons grown in microfluidic channels. **(C)** The response of metal–oxide memristive device to spiking activity recorded in the culture. Black line—voltage drop on memristor, red line—voltage drop on load resistor as current sensor, and blue line—resistance of memristive device responding in a volatile or non-volatile manner to noise and spikes with different parameters. **(D)** The example of CMOS integration of metal–oxide memristive device based on thin ZrO_2_(Y) film sandwiched between top metal layers of CMOS circuit. **(E)** The typical diagram of registration, amplification, and analysis of bioelectric activity by using multielectrode/memristive arrays and embedded CMOS circuits. **(F)** The typical spiking neural architecture with competitive interneuron connections. **(G)** The scheme of extracellular electrical stimulation of living neurons modulated by the electronic subsystem to control their activity.

Figure 1A demonstrates a schematic representation of the proposed neurohybrid system, which consists of several functional layers combined in one CMOS-integrated chip. The top layer is a part of the neuronal system represented here by a culture of dissociated hippocampal cells grown on multielectrode array (MEA) and functionally ordered by a special layout of microfluidic channels indicated in Figure 1B. The MEA is used for extracellular registration and stimulation of neurons *in vitro* and is implemented on the top metallization layers of the CMOS layer together with an array of memristive devices (Figure 1D). The simplest task performed by memristive devices is the direct processing of spiking activity of the biological network (Figure 1C); however, self-learning neural network architectures based on fully connected cross-bar memristive arrays can be designed for adaptive decoding of spatiotemporal characteristics of bioelectric activity. The output of this artificial network (Figure 1F) can be used to control the cellular network via gradual modulation of extracellular stimulation (Figure 1G) according to the given protocol. This way, analog and digital circuits should be implemented in the main CMOS layer (Figure 1E) for accessing and controlling the MEA and memristive devices, amplifying, generating, and transmitting signals between layers. To create neurohybrid chip, joint design and optimization are required for all mentioned elements at the levels of materials, devices, architectures, and systems. Within the framework of this concept, the following subjects of interrelated research and developments should be considered at fundamental and applied levels:

1) Neural networks cultured *in vitro* with a given connectivity to implement a certain information function;
2) Microfluidic cell manipulation techniques on a chip;
3) CMOS- and bio-compatible technology for the MEA fabrication;
4) Scalable and CMOS-compatible memristive devices;
5) Microelectrode and memristive arrays integrated on-chip with CMOS electronics;
6) Analog/digital peripheral and control circuits on CMOS chip;
7) Specialized SNN based on memristive arrays and CMOS electronics;
8) Interconnection/integration solutions for connecting various functional modules.

The first two tasks are required only in the case of creating a neurohybrid device like the neuroanimat, with information processing by an ensemble of cultured living neuron cells. To create both implantable and non-implantable devices such as neuroprostheses, the implementation of this route apparently should start with the task 3.

Two main groups of challenges must be addressed for the successful development of this technology. From the biological side of neural integration, the main problems are related to biocompatibility and matching mechanical properties of MEA materials in contact with neuronal culture, device geometries and accessibility to neuronal culture, their scaling to brain activity *in vivo*, as well as the reaction of living neurons to electrical stimulation and power dissipation (including glial scarring). From the electronic engineering side, we should note the required high spatiotemporal resolution of MEA, transition from 2D to 3D electrode system, minimum size and high density of memristive devices needed for subsequent monolithic integration, area- and energy-efficient solutions for analog information processing by memristive circuits. Both groups of challenges, possible solutions, and trade-offs are considered in the corresponding sections below.

## Living Neural Network: Biological Side of Neural Integration

The main problem of neuronal cultures *in vitro* is related to homogeneous network structure, which is developed in randomly patterned cells on the substrate. During the last decade, new methods of neuroengineering have been developed to control the position of cells and direction of axon and dendrite growth (le Feber et al., 2015; Na et al., 2016; Renault et al., 2016). Recently, it has been shown that the main feature of functional network topology as unidirectional synaptic connectivity between cell clusters can also be engineered using microfluidic technology (Gladkov et al., 2017; Poli et al., 2017; Forró et al., 2018). Being implanted in the damaged brain, such tools of network structure manipulation allow one to mimic brain areas, which are involved in reflex activity, pattern retrieval in multilayered unidirectional network (Brewer et al., 2013; Poli et al., 2017) for neural tissue recovery from brain injury (Shimba et al., 2019). Next, it could be combined with an array of non-invasive planar microelectrodes, which provide spiking activity registration and stimulation of isolated or multiple neurons. Spiking activity could be monitored and induced in several independent axonal pathways, which grow between subnetworks through the microchannels. Thus, the precise input and output could be implemented in engineered multilayered network with the designed connectivity, where the full potential of the proposed task can be solved in closed-loop conditions with memristive spiking network. First, such a system could be used for the stabilization of spontaneous activity, which slowly stochastically changes, and second, to classify patterns according to various input signals and induce spike-timing-dependent plasticity (STDP) in a living network, where pre- and postsynaptic neurons could be accessed independently.

Biocompatibility and mechanical matching of materials are the key problems that arise on the way to neural integration. They have been already addressed in many commercial MEA by using gold, platinum, indium tin oxide (ITO), and titanium nitride (TiN) as electrode materials. The signal-to-noise ratio (SNR) depends strongly on the biological part of the system, but can be increased by the small impedance of recording electrodes. In order to reduce the impedance and increase the charge transfer efficiency, the surface area of electrodes can be modified by covering with porous conductive materials, such as Pt-black, Au nanoparticles, carbon nanotubes (CNTs), and conductive polymers like poly(3,4-ethylenedioxythiophene) (PEDOT) (Obien et al., 2015). Moreover, the enhanced biocompatibility has been demonstrated for electrodes with a nanostructured porous surface in the form of laser-micropatterned PEDOT:PSS (Santoro et al., 2017). The next level of improved compatibility between electrodes and cells or living tissue relies on the use of extracellular matrix materials, which increase the adhesive properties of the electrodes and reduce the risk of inflammatory processes (Won et al., 2018).

An important problem of the registration of neuronal activity is associated with the geometry/topology and spatial resolution of microelectrode arrays. Conventional MEAs do not allow recording the activity of individual cells, because the step between electrodes (>30 μm) exceeds the neuron soma size (about 12–18 μm). Owing to the advanced CMOS technology, a new type of MEA has been commercialized, in which amplifiers and ADC are located on one chip with electrodes. This approach reduces the inter-electrode distance and consequently increases the spatial resolution of electrodes. The search for optimal solutions to combine high spatial resolution with a high SNR is currently underway (Ghane-Motlagh and Sawan, 2013; Müller et al., 2015). The proposed system concept presumes a 2D neuron interface on top of the MEA electronics. However, planar electrodes reach their limits when it comes to tissue slices or cell clusters. Although, a 3D-MEA with micron-size electrodes penetrating 40–100 μm deep into the tissue is already on the market^1^, the lattice-like 3D electrode interface should be developed to really mimic or interface the brain.

All these problems are exacerbated when scaling the proposed technology to registration and stimulation of brain activity *in vivo*, especially taking into account high conductivity, inertness, biocompatibility, and stretchability required for the interaction with living tissue (Qi et al., 2017). Devices for detecting neural activity *in vivo* can be fabricated in the form of 2D or 3D arrays of electrodes combined on one substrate. Two types of 3D probes are widely used: electrodes placed on an array of silicon needles and neural probes, on which arrays of electrodes are located. Recently, densely arranged probes based on silicon-on-insulator (SOI) technology have been actively developed (Scholvin et al., 2015; Angotzi et al., 2017; Lopez, 2019). The dense arrangement of electrodes allows spatial oversampling of neural activity and accurate sorting of spikes. In the active neural probes, local amplification of the recorded signal near the electrode with microfabricated CMOS circuit improves the recording quality by reducing the electrode impedance and crosstalk between neighboring shank wires (Raducanu et al., 2017). Simultaneous recording of signals from a large number of electrodes (up to 1400) can be possible due to the time division multiplexing method. In addition, when developing neural probes, it is necessary to consider heating of tissue due to power dissipation, which is limited to a threshold of 1°C for chronic experiments (Kim et al., 2007). To reduce tissue damage and inflammatory response, the size of the shanks should be miniaturized and can reach 25 μm for the SOI technology. For the passive probes, one of the approaches to thin the shank can be based on materials with low stiffness like nanoelectrode filaments or poly(etherimide) fibers. On the other hand, such materials require additional support during implantation, particularly, control of localization and speed modes of insertion (Dryg et al., 2015), as well as special guides from soluble materials (Won et al., 2018).

Another important problem is the reaction of living cultures and tissues at the interface with the artificial electronic subsystem. Needless to say that purely electrical contact can serve non-invasively not affecting the cell in contrast to different methods of optical recordings or chemical manipulations. More complicated task is to provide correct stimulation of the target area in the brain or in the neuronal culture. Problems may appear in long-term implantations, when the neuronal system under stimulation (including electrical one) starts to adapt itself maintaining the network homeostasis and trying to escape the external perturbation (Middleton et al., 2010; Graczyk et al., 2018). Adaptation is based on the mechanism of homeostatic plasticity, which ensures the functional stability of neuronal system by equipoising intrinsic excitability and synaptic strength. It balances the network excitation and inhibition, and coordinates the changes in circuit connectivity (Tien and Kerschensteiner, 2018). In addition, any mechanical impact on the brain tissue, such as implantation of electrodes, may cause the appearance of a glial scar that restricts the area damaged by the electrodes. For instance, sharp electrodes implanted into a brain after some time are insulated by glial cells produced around the electrode hence decreasing the effect of stimulation (Sillay et al., 2013; Wu et al., 2013). The gliotic encapsulation problem can be mitigated by chemical functionalization of materials at the electrode–tissue interface. Coating of electrodes with extracellular matrix proteins, collagen and Matrigel films can reduce the astrogliotic scarring (He et al., 2006; De Faveri et al., 2014; Shen et al., 2015). Another efficient approach to mitigate the rejection is to reduce the electrode size, for example, by using the PEDOT-coated carbon fiber as a material of electrodes (Patel et al., 2016).

The limiting factor to resolution and functionality of the proposed neurohybrid concept may be the power consumption needed for a given SNR when basing the concept on CMOS (or any other semiconductor) technology. This problem depends on the type of interface with the neuronal system (*in vitro* or *in vivo*) and the energy efficiency of electronic subsystem. In the case of *in vitro* interface, the existing commercially available CMOS MEA has the overall power consumption of about 30 W^2^, which is mainly determined by the off-chip interface electronics and does not include the data processing and analysis equipment. The *in vivo* interface systems have been studied previously in relation to neural prostheses for restoring and enhancing memory (Berger et al., 2011; Hampson et al., 2018; Song et al., 2018) also by using PC-controlled multichannel recording/stimulation closed-loop systems and special mathematical models. To the best of our knowledge, none of the mentioned systems has been implemented yet on a single chip. Although miniaturization is a general requirement to create such bioelectronic platforms (Birmingham et al., 2014), we believe that it can only be achieved using the area- and energy-efficient memristive electronics based on CMOS technology and shown below. Of course, this task should be reached hand in hand with the development of miniaturized wireless systems for energy harvesting and bi-directional communication that will definitely improve safety, access to anatomical sites, and enable ultra-minimally invasive delivery methods, reducing tissue trauma during implantation and immune response (Masius and Wong, 2020; Piech et al., 2020).

## Memristive Devices: Toward Cmos Integration

A memristor (memory resistor) was predicted by Chua (1971) as the fourth passive element of electrical circuits. For a long time, it was considered as a theoretical object. Only in 2008, the memristive effect was first correlated (Strukov et al., 2008) with the phenomenon of reversible resistive switching, which can occur in a simple thin-film metal–oxide–metal nanostructure and is associated with local rearrangement of the oxide atomic structure and composition under the action of inhomogeneous electric field, temperature, and concentration gradients (Ielmini and Waser, 2016). Currently, memristors and memristive systems are the basis of a new paradigm in electronics related to creation of brain-like network architectures by using the ability of memristive devices to emulate the most important functions of biological synapses and neurons. Since 2015, there has been an increase in the number of publications regarding a hardware implementation of the simplest artificial neural networks (ANNs) (most often in the form of a single-layer perceptron) based on a limited number of memristive connections (Prezioso et al., 2015; Serb et al., 2016; Yao et al., 2017). Larger integrated memristive 1T-1R or passive cross-bar arrays have been fabricated and shown to date (Cai et al., 2019; Kataeva et al., 2019; Zhou et al., 2019) to implement various multiplication operations and neuromorphic functionality on the basis of precise analog tuning the conductance of memristive devices. Although some higher functionalities of board-integrated systems like multilayer perceptron (Bayat et al., 2018; Li et al., 2018a; Mikhaylov et al., 2018) and the first fully memristive neural network with unsupervised learning (Wang et al., 2018) were demonstrated and revolutionized, the higher functionalities are still restricted with a practical size up to 64 × 128 of memristive arrays.

Thus, the necessary condition for the development of advanced functional electronic circuits based on memristors is their integration with mixed analog-digital CMOS transistor circuits. At the same time, the capabilities and functionality of traditional ANN architectures based on programmable memristive weights are limited by the size of the memristive array, the increase of which is constrained not by low scalability (the minimum size of the memristive element may be of the order of nanometer; Pi et al., 2019), but by insufficient reproducibility of device parameters due to the stochastic nature of resistive switching. For example, the widely used back propagation updating rule, which has been proved to be efficient for traditional supervised neural networks, often requires additional write-verification techniques (Yao et al., 2017) to modulate memristive devices into the desired states, incurring software/hardware overheads on memristive neurohybrid architectures.

The non-linear behavior of memristive devices in response to electrical pulses together with their unique scalability are the most important advantages that determine a unique possibility of hardware implementation of SNN (Demin and Nekhaev, 2018; Guo et al., 2019) based on the processes of self-organization in neural network architectures and qualitatively different from traditional neural networks (perceptrons). We believe that implementation of brain-like networks of future generations will be based on the stochastic dynamics of memristors and synchronization of neural oscillators. Such works are carried out at the most basic level (Ignatov et al., 2016; Gerasimova et al., 2017), demonstrate the possibility of implementing higher (cognitive) brain functions, but require the development of adequate models of neural synchrony based on stochastic memristive plasticity.

Nevertheless, such a rapid progress in the implementation of memristive neuromorphic systems makes it possible not only to expect in the nearest future the creation of brain-like networks with memristive plasticity for novel computing paradigms, but also to take the next step and develop memristive neurohybrid systems on the basis of intrinsic analogy in the properties of memristive and natural systems. It is important to note that compact memristor-based devices for real-time processing of bioelectric activity (threshold detection of spikes) can be created owing to the integrative change in their resistive state (Gupta et al., 2016). In this case, the metastable (volatile) behavior is an important property of memristive devices for continuous and energy-efficient encoding of large volumes of spiking activity of living biological cultures (Gupta et al., 2018). It should be mentioned that effective use of memristors in neurohybrid systems is dependent on the predictable behavior of memristive nanomaterials and devices, as well as on the ability to control the parameters of their non-linear response to complex electrical signals, which should be a subject of comprehensive study at the micro- and macro-levels.

Noise plays a very significant and constructive role in memristive devices, and only recently new investigations on the positive role of noise have been started (Mikhaylov et al., 2016; Filatov et al., 2019). Nowadays, there are many known examples, where the interplay of non-linearity and fluctuations can change the properties of a stochastic system in a counter-intuitive way, in classical and quantum physics (Fiasconaro et al., 2004; Chichigina et al., 2005, 2011; Valenti et al., 2008, 2015; Falci et al., 2013; Spagnolo et al., 2015, 2016, 2018a,b). Furthermore, internal and external noise can play a positive role in the switching dynamics of memristors, as in stochastic resonance phenomenon (La Barbera and Spagnolo, 2002; Valenti et al., 2004; Agudov et al., 2010). This paves the way for using the intensity of fluctuations as a control parameter for switching dynamics in memristive devices (Agudov et al., 2020).

## Cmos Circuits: On-Chip Analog and Digital Systems

As noted above, a significant progress has been demonstrated on the way toward integration of memristive arrays and CMOS circuits (Cai et al., 2019; Kataeva et al., 2019). The electronic subsystem required for the CMOS integration of memristive arrays includes peripheral and control circuitry. In Kataeva et al. (2019), large passive memristive cross-bars are accessed via on-chip CMOS interface circuits which are controlled by a custom FPGA board. To reduce latency and power consumption, a full set of mixed-signal interface blocks and a digital processor have been recently integrated together with memristive cross-bar array on a single chip (Cai et al., 2019), instead of using discrete components on printed board. On-chip integration of processor allows the neuron functions and network structures to be reprogrammed through simple software changes, enabling different algorithms to be mapped on the same hardware platform.

With respect to the electronic subsystem of the neurohybrid chip, a number of technical problems have to be solved to organize the optimal interaction of living neuronal culture with memristive arrays. Reading, processing, and reflection of the spiking activity of neural cells must be carried out with a duration of no more than a typical pulse (spike) in the areas of the neurons. At the prototyping stage, a separate reading amplifier and recording amplifier cannot be allocated to each electrode of contact with a living culture due to the limited area of the CMOS layer. This should be implemented in future with higher design standards or a smaller number of electrodes.

It is necessary to implement an array of reading and writing amplifiers in the CMOS layer, which allows transmitting pulses from a living culture through electrodes to memristive array and vice versa, simultaneously on a certain surface area. The reading and writing amplifiers must be tuned to the signal from living tissue amplified to the levels of active operating modes of memristors. In the CMOS layer, it is also necessary to implement access circuits for electrodes and memristors at row and column addresses.

Circuits for stimulation of living culture/tissue (by using the response of memristive network) are supposed to be implemented on the basis of the pulse-width modulation (PWM). If necessary, for the simultaneous reading of the electrode states in the CMOS layer, banks of buffer memory can be implemented. It is proposed to use ADC and DAC circuits to input and output information about the analog state of memristors, but the required bits of the ADC and DAC should be determined at the prototyping stage (8 bits are assumed in the layout). The initial input and subsequent output of information for a set of statistics on the experiment and processing can be implemented on the basis of standard bidirectional interfaces (Cai et al., 2019).

Although the local resistive switching effect in memristive devices provides the unique compactness, fast and energy-efficient operation of passive memristive arrays (Xia and Yang, 2019), the active arrays integrated with peripheral and control electronics should be always a subject of explicit evaluation and benchmarking depending on the development/prototyping stage (Cai et al., 2019; Zhao et al., 2020). Recently, several reports on such benchmarking have shown potential advantages of memristive chips over conventional ones: 19.7, 6.5 times, and 2 orders of magnitude better energy efficiency compared to the Google’s tensor processing unit (TPU), a highly optimized application-specific integrated circuit (ASIC) system, and the state-of-the-art graphics-processing unit (GPU), respectively (Sun et al., 2020; Yao et al., 2020). The performance benchmark of memristive neuromorphic computing system shows 110 times better energy efficiency and 30 times better performance density compared to Tesla V100 GPU. So, even rough estimates for memristive circuits considered in this article allow one to imagine their great potential from the viewpoint of speed, performance, power consumption, and compactness.

The issue of reliability of memristive neural networks is also currently in the eyeshot of many researchers and requires the use of system approach and comprehensive consideration (see Zhao et al., 2020 for review on the status of reliability studies in this field). An example of such a system approach to ensure the reliability of neural networks based on memristors is proposed by the authors (Danilin et al., 2019; Shchanikov et al., 2020). Another promising way is to use specialized algorithms for tuning (training) memristor-based neural networks, as it is proposed in Wang et al. (2019). This approach makes it possible to create a neural network that self-adapts to non-idealities of the 1T-1R memristive array, thereby providing the necessary level of reliability.

One more important limitation when creating electronic devices in contact with living cultures/tissues is to preserve a trade-off between performance and power dissipation. On-chip processing is more efficient than transmitting raw data to the external processing unit (Zhuk et al., 2020), but the power consumption of state-of-the-art digital processors is too high. The dissipated power of memristive chips, according to the estimates made by a number of research groups, does not exceed tens of mW: 13.7 mW (Li et al., 2018b), 7.438 mW (Yao et al., 2020), 6.62 mW (Wang et al., 2019), 42.1 mW (Lee et al., 2020), 64.4 mW (Cai et al., 2019). The power dissipation strongly depends on the amplitudes and frequencies of the signals and increases with increasing the values of these parameters, which is not necessary in principle when working with living neurons. In addition, in a traditional computing system, power is also dissipated in memory units (Horowitz, 2014) and even much more in data movement, while both data storage and computation can be combined in one memristive device. So, the use of memristors to create a system on chip seems to be much more efficient and safe for neural interfacing.

Therefore, one can argue that memristive CMOS circuits will outperform traditional digital computing tools (CPU, GPU, TPU, ASIC) in all key parameters for a wide range of data-intensive applications, one of which is the real-time on-chip processing of electrophysiological data in the frame of the proposed neurohybrid concept.

## Memristive Neural Arcitectures: Toward Neuroprosthetics

Biological relevance should be ensured when developing substitutive (neuroprostheses, motorized prostheses) and assisting neuromorphic systems [computer interfaces (Lobov et al., 2016), exoskeletons (Mironov et al., 2017), wheelchairs, “neuromobiles” (Mironov et al., 2018)]. Here, if possible, the same neural “language” and the same principles of information processing should be used as in a biological brain. Only in this case, over time, we can expect the blurring of the boundary between living and artificial neural subsystems, which will ultimately lead to the expansion of human capabilities. On the other hand, in all the neurochip perspective applications discussed here, we have arrays of implantable or non-invasively attached electrodes that record in real time the electrical activity of ensembles of neurons and/or muscle fibers. It is clear that the more electrodes and more frequently the signal is taken from each of them, the higher is the spatial (topographic) and temporal resolution and, accordingly, the potentially higher is the accuracy of sensory recognition (vision, hearing) or motor control commands sent to an electromechanical prosthesis. In this manner, we get a huge amount of data that needs to be processed in real time. Currently, it is common to use an external processor, which performs this processing and provides an interface between an external part of a prosthesis (camera, microphone, artificial limb) and the microcontroller device from a living tissue side. However, the solution of such problems could be strongly optimized by exploiting a highly specialized processor with neural network architecture adapted for this specific kind of calculation and serving as if it is a natural extension of the biological nervous system (Boi et al., 2016). In this case, the computing device would be capable of processing a large input dimension (determined by the number of electrodes in the MEA) and performing the required real-time signal processing. In our opinion, the SNN architecture based on phenomenological models and integrated into the proposed hybrid system seems to be a good compromise in the sense of both biological similarity and computational/power cost.

Recently, the first steps have been taken toward EMG (Lobov et al., 2015, 2020a) and EEG (Goel et al., 2006; Tahtirvanci et al., 2018) interfaces based on SNNs. However, until now, no learning rule for SNNs has been proposed, which is equal in its universality and effectiveness to the back propagation algorithm for ANNs based on formal neurons. Several attempts were made to adapt the “backprop” and its variations to SNNs (Hong et al., 2010; Xu et al., 2013; Esser et al., 2016), but associative learning based on synaptic plasticity similar to that for living neurons seems to be a more “natural” way. Indeed, traditional formal neural networks contain artificial neurons with a static activation function as key computational elements, i.e. there is no dynamics in such systems. Consequently, such systems are quite difficult to synchronize with time sequences of individual spikes recorded in a biological nervous system. Spiking artificial neurons, as well as their biological prototypes, generate spike sequences that could be synchronized with the biological pulse signal through a non-linear interface—an artificial analog of synapse, a memristor. Namely, it has been recently shown that a system consisting of several spiking pre-synaptic neurons connected via memristive devices to the one post-synaptic neuron can adapt their conductivities (synaptic weights) to the same distribution under STDP updates by the repeatable pre- and post-synaptic trains of pulses, independent of the initial resistances of memristors or their device-to-device variability (Emelyanov et al., 2020). This means that the non-linear memristive spiking system memorizes only useful information about millisecond-scale time intervals between spikes which could encode some real data about perceived objects from an environment or motor commands to actuators. In a neurohybrid interface, spikes from biological neurons could be transformed online (synchronized) to the trains of voltage pulses generated by artificial spiking units which, in turn, could be used for the informative update of memristive weights as described above.

Neural networks of a living brain appear to use both temporal and frequency coding (Clopath et al., 2010; Masquelier and Deco, 2013). Similar behavior is observed in memristive devices based electronic circuits (Battistoni et al., 2019a). Thus, the process of SNN learning should provide both types of coding. In addition, SNNs, unlike their formal counterparts, can be trained by bio-plausible, so-called local rules of synaptic weight change using information only about the activity of interconnected neurons and synaptic efficacy (weight magnitude) between them. These rules do not require information from the outside, as in the case of learning by error back propagation technique, and therefore can be the basis of self-learning computing systems, with a change in synaptic weight according to the rules of the Hebbian (Morris, 1999), STDP (Bi and Poo, 1998), BCM (Bienenstock et al., 1982), metabolic (Yousefzadeh et al., 2018), or homeostatic (van Rossum et al., 2000) types. In the case of frequency coding, it is necessary to use frequency-dependent varieties of STDP, such as the triplet-based rule STDP (Pfister and Gerstner, 2006) or voltage-based STDP (Clopath et al., 2010). Recent studies have shown the possibility of rate and temporal coding in SNN using a combination of Hebbian learning (through triplet-based STDP), synaptic and neuronal competition (Lobov et al., 2020a, b). Hebbian and other STDP rules have been demonstrated for a large number of different kinds of memristors (Kim et al., 2015; Ielmini and Waser, 2016; Emelyanov et al., 2019; Minnekhanov et al., 2019) that confirms their high potential to serve as the self-adjusting weights between neurons in SNN.

Moreover, on the basis of spiking architectures, it is possible to naturally train recurrent networks in which there are feedback connections from deeper layers of neurons to less deep layers, as well as the lateral connections between neurons of the same layer (Demin and Nekhaev, 2018). In general, such architectures cannot be reduced to a feedforward neural network, such as a long-short term memory (LSTM) “unrolled” in several consecutive modules (Hochreiter and Schmidhuber, 1997; Brownlee, 2019). Therefore, recurrent SNNs can potentially be trained on the basis of local rules to realize complex dynamic patterns corresponding to those in the biological part of neurointerface. This kind of training can take place in real time, continuously adapting to the individual characteristics of a user’s behavior. This is a practically inaccessible task for the formal ANN architectures that require *a priori* training by error back propagation on a set of pre-recorded patterns.

Implementation of hardware SNN architectures based on memristors certainly requires additional wide studies: first, to identify a minimum set of local learning rules sufficient for the convergence of training the network to a solution of a given problem, second, to seek for the possibility of adapting local rules (like that of STDP type) to hardware implementation with memristive elements (either by appropriate selection of the memristive material or by engineering the temporal sequence and/or shape of spikes generated by artificial neurons), and, at last, to optimize (by energy efficiency, area and computing performance) the SNN architecture design and placing corresponding periphery systems on neurohybrid chip under development. Although the higher computational/power efficiency of SNN is one of the well-known advantages over traditional neural architectures (Lee et al., 2020), further improvement in this direction can be based on rich dynamics of memristive devices and avoiding special programming circuitry used for the implementation of learning rules.

The most interesting direction at the boundaries of neurotechnology and neuromorphic prosthetics has recently emerged thanks to the seminal paper (Juzekaeva et al., 2019), where the main principles and feasibility of a memristive prosthesis of a synapse connecting two not connected via natural synapses neurons of a rat brain slice are proposed. This work triggered the discussion of the option to use stochastic memristive devices of different nature as main building block of neuromorphic prosthesis relocating functions and topology of natural neuronal circuits. Some steps in this direction have been already presented (Talanov et al., 2018b) including blueprints of a memristive neuron circuit (Talanov et al., 2017a, b, 2018a). As the number of memristive neurons available grows and the technology of their fabrication becomes more and more mature, we could expect the rise of the number of spiking solutions for the reimplementation of neuronal structures as electronic memristive circuits with more and more bio-plausible functions. Possibly, the most promising and timely problem, due to the lesser number of neurons and synapses, is the spinal cord direction that seems to attract rising interest of the researchers community (Gill et al., 2018; Wagner et al., 2018). The current state of neurorehabilitation of patients with complete spinal cord injury including epidural spinal cord stimulation is mainly experimental (Lavrov et al., 2008; Gad et al., 2013; Moraud et al., 2016), and it seems that a memristive implementation of part of the spinal cord circuits could restore the walking patterns of patients with complete SCI. We should not limit ourselves with the reimplementation of the part of the nervous system for patients, we could envision the further development of augmented nervous systems with digital extensions using memristive properties of self-adaptation for the bidirectional brain to machine interfaces (Musk and Neuralink, 2019) based on the proposed neurohybrid chip approach.

## Conclusion and Outlook

Here the concept of a single neurohybrid chip is proposed based on existing and future solutions in the field of neural cells and microfluidic technologies, which allow spatial structuring of living neural network combined with CMOS MEA and memristive arrays for real-time recording, processing, and stimulation of bioelectric activity interfaced and controlled by mixed analog–digital circuits on the same chip. This concept paves the way toward the creation of compact biosensors and neuroprosthetics that cannot be realized on the basis of traditional neurointerface architectures. The functionality of the proposed neurohybrid chip is limited in several domains on the side of electronic subsystem, including challenges associated with power consumption and reliability of memristive circuits. Significant efforts should be made to further understand the basic principles of learning in living neural networks and development of universal learning algorithms for SNN, providing their biological relevance and compatibility with memristive arrays. The key challenge on the road toward neurohybrid systems still remains in the reliable interaction between living neurons and electronics. Although memristors can provide efficient recording and on-chip processing of the neural activity, a number of problems are still related to biocompatibility and mechanical impact, geometry, placement, and miniaturization of electrodes and probes, as well as the reaction of living cultures and tissues at the interface with the artificial electronic subsystem. The potential transition from the proposed 2D to the 3D electrode system could provide some solutions, opening further questions related to implantation into deeper regions of the brain without causing structural damage to the tissue.

We hope that future realizations of the proposed concept may go beyond the CMOS limitations and rely on a direct synapse-scale interface based on organic and stochastic memristive nano-networks. Such nano-neurointerface should provide network distributed stimulation, when each stimulation event will be at the level of small synaptic currents of physiological range, hence not affecting the self-protection mechanisms of the brain. Designed this way, recording, processing, and stimulation electronic networks can be “physiologically” integrated into different brain areas to compensate or enhance brain functions from sensory level to the level of cognition and memory. Integrated into neural tissue memristive networks can also shed the light on the fundamental questions of analog neuron information processing.

**FIGURE 2:**
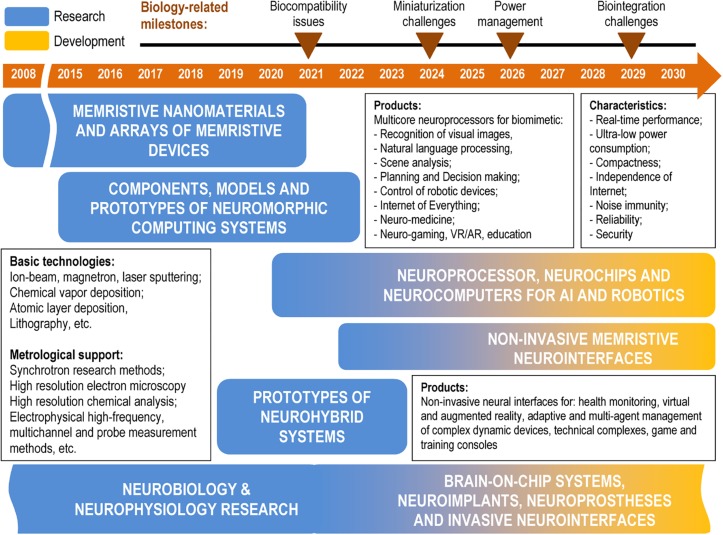
A roadmap of memristive neuromorphic and neurohybrid systems.

To illustrate the proposed approaches and related products in a foreseeable timeline, Figure 2 shows a roadmap of memristive neuromorphic and neurohybrid systems focused on the specialized hardware based on the architecture and principles of biological neural networks to support the development and mass introduction of artificial intelligence technologies, machine learning, neuroprosthetics, and neural interfaces. The roadmap starts tentatively in 2008 with the beginning of the current wave of interest to memristors (Strukov et al., 2008) and includes long-lasting research in the broad fields of neurobiology and neurophysiology. The following product niches are provided at different stages of development in this roadmap:

1) Neuromorphic computing systems;
2) Non-invasive memristive neurointerfaces;
3) Neuroimplants, neuroprostheses, and invasive neurointerfaces.

There are the unique properties of memristive devices that determine their decisive importance in the development of neuromorphic and neurohybrid systems for computing systems, brain–computer interfaces, and neuroprosthetics. These products will occupy a significant part of the global high-tech market worth trillions of dollars by 2030, taking into account the speed of development and implementation of artificial intelligence technologies, the Internet of Things, technologies of big data, smart city, robotics. Targets of the near future are neuroprosthetics, instrumental adjustment/support/enhancement of human sensing and cognitive abilities.

Hardware support is not just necessary for these technologies—the further development of neurocognitive technology industry and artificial intelligence is impossible without it due to the pronounced inadequacy of the traditional von Neumann architecture of computers for solving anthropogenic problems requiring a neural network architecture. As a result, we have unsatisfactory performance with huge energy consumption by the existing ICT infrastructure in the processing of even current (ongoing) anthropogenic tasks. This trend with the spread of intelligent technologies will only worsen, and therefore the development of specialized hardware of neuromorphic and neurohybrid systems (discussed here and based on memristors in a priority) is a key condition for the development of high-tech industries as a whole.

The development of artificial hardware systems should be in line with the bio- and neurotechnologies shown on the roadmap in the form of critical milestones, when a clear decision should be made on the most appropriate solutions. Over the past two decades, some progress has been observed in the development of biocompatible materials with the aim of creating multi-channel recording devices for neuronal networks activity both *in vitro* and *in vivo*. The prototypes of such devices are already implanted in the brain of animals for a long time with minimal immune response. Further optimization involves minimizing damage during implantation into the brain by reducing the size and increasing the flexibility of the probes in conjunction with the electrodes scaling. At the same time, the development of new-generation neuorohybrid systems will require a lot of special not yet obtained tools and preliminary experiments on animal models. These are the further advancement of neural interfacing (in view of microelectrode biocompatibility, reliability and rejection problems, 2D to 3D transition, etc.), chronic neural pattern recognition and control devices (neurochips and algorithms for them), power management, signal processing, and data transfer in miniaturized platforms. Also, there is a problem with proof-of-principle investigation of which of neural circuits influence over disease progression in representative animal models. All these stages are crucial for the development of invasive neurointerfaces and memristive control systems for them. These neurobiology and neurophysiology involved investigations are still in progress and, due to the vast literature reports, could begin to develop mass products in 2 or 3 years.

## Data Availability Statement

The datasets generated for this study are available on request to the corresponding author.

## Author Contributions

All authors gave substantial contribution to the development of this work equally, drafting, and revising it critically. Furthermore, all authors approved its final version for publication.

## Conflict of Interest

The authors declare that the research was conducted in the absence of any commercial or financial relationships that could be construed as a potential conflict of interest.

## Footnotes

1 https://www.multichannelsystems.com/news/3d-meas-recording-inner-cell-layers

2 http://www.multichannelsystems.com/sites/multichannelsystems.com/files/documents/manuals/CMOS-MEA5000-System_Manual.pdf
